# Posttraumatic Stress and Posttraumatic Stress Disorder after Termination of Pregnancy and Reproductive Loss: A Systematic Review

**DOI:** 10.1155/2015/646345

**Published:** 2015-02-05

**Authors:** Viltė Daugirdaitė, Olga van den Akker, Satvinder Purewal

**Affiliations:** ^1^Department of General Psychology, Philosophy Faculty, Vilnius University, Universiteto 9/1, Vilnius, LT-01513, Lithuania; ^2^Department of Psychology, Middlesex University, The Burroughs, Hendon, London NW4 4BT, UK; ^3^Institute of Psychology, University of Wolverhampton, Wulfruna Street, Wolverhampton WV1 1LY, UK

## Abstract

*Objective*. The aims of this systematic review were to integrate the research on posttraumatic stress (PTS) and posttraumatic stress disorder (PTSD) after termination of pregnancy (TOP), miscarriage, perinatal death, stillbirth, neonatal death, and failed in vitro fertilisation (IVF). *Methods*. Electronic databases (AMED, British Nursing Index, CINAHL, MEDLINE, SPORTDiscus, PsycINFO, PubMEd, ScienceDirect) were searched for articles using PRISMA guidelines. *Results*. Data from 48 studies were included. Quality of the research was generally good. PTS/PTSD has been investigated in TOP and miscarriage more than perinatal loss, stillbirth, and neonatal death. In all reproductive losses and TOPs, the prevalence of PTS was greater than PTSD, both decreased over time, and longer gestational age is associated with higher levels of PTS/PTSD. Women have generally reported more PTS or PTSD than men. Sociodemographic characteristics (e.g., younger age, lower education, and history of previous traumas or mental health problems) and psychsocial factors influence PTS and PTSD after TOP and reproductive loss. *Conclusions*. This systematic review is the first to investigate PTS/PTSD after reproductive loss. Patients with advanced pregnancies, a history of previous traumas, mental health problems, and adverse psychosocial profiles should be considered as high risk for developing PTS or PTSD following reproductive loss.

## 1. Introduction

Posttraumatic stress (PTS) and posttraumatic stress disorder (PTSD) after reproductive loss have not been well recognised, despite the growing documentation of adverse psychological states associated with reproductive losses. Our focus is on PTS and PTSD but did not include acute stress disorder (ASD) because ASD is a separate disorder diagnosed only in the first month following the traumatic event. Although the classification of TOP and reproductive loss varies from country to country [1], TOP broadly refers to the* termination* of a clinical pregnancy and miscarriage to the* spontaneous loss* of a clinical pregnancy before 20 completed weeks of gestation. Perinatal death, on the other hand, refers to a fetal or neonatal death after 20+ weeks during pregnancy and childbirth or up to 7 days after birth, whereas stillbirth denotes the death of a (20+ weeks of gestational age) baby before the complete expulsion/extraction from its mother. A neonatal death is said to have occurred when a live born baby dies within 28 days of birth [2]. Failed in vitro fertilisation (IVF) is also considered by some infertile couples as a reproductive loss [3], with some women reporting grief, sadness, and distress with IVF failures [4, 5] and, for those who do become pregnant, a more intense protective attachment to their fetus [6]. PTS and PTSD can evolve after any of these reproductive losses [7–10].

Further, TOP is different from other reproductive losses as it involves a “choice” of the woman to terminate a pregnancy or not, but the event itself is a stressful situation and can become traumatic for some women [11]. It is also important to separate nonmedical TOP (which is usually requested for social reasons) from medical TOP, which is usually requested when there is evidence of foetal abnormality which could lead to giving birth to a baby unlikely to survive long, or when the difficulties of rearing an affected child are perceived as too great to be acceptable to the couple [12]. The differences between medical and nonmedical TOP are further highlighted in societal/cultural acceptance and legal status. For instance, many African or Latin American countries will not allow nonmedical TOPs [13], although the World Health Organisation [14] reported that highly restrictive abortion laws did not lower TOP rates, stigmatising this illegal practice further. Specifically, the abortion rate is 29 per 1,000 women of childbearing age in Africa (where TOP is illegal under most circumstances in most countries) compared with 12 per 1000 in Western Europe (where abortion is permitted in many countries). Nonmedical TOP may not be recognized as a traumatic event by some women because they do not want the baby [15]. However, some of these women subsequently regret having had the abortion and can experience it as trauma [16]. Not all women experiencing a nonmedical TOP will experience posttraumatic consequences [17] but that is also true for medical TOP and all other reproductive losses. Nonetheless the psychological impact of nonmedical and medical TOP may be different, but the extent to which they differ needs further investigation.

Although mental health promotion following reproductive loss is underinvestigated [18], PTSD after childbirth and pregnancy loss has been distinguished from postnatal depression and complicated grief [19, 20]. Studies which have investigated PTS and/or PTSD following reproductive loss and TOP have reported mixed results [13, 21]. For example, high levels of PTSD following nonmedical TOP have been reported by some [22] but not all previous research [23]. At present, there is no systematically pooled research evidence on PTS and its disorder after TOP and reproductive loss. The rationale for this systematic review is therefore to reconcile previous research and deliver the first review that integrates research on PTS and PTSD after all reproductive losses (TOP, miscarriage, perinatal loss, stillbirth, neonatal death, and failed IVF) for women and men and investigate the prevalence and factors influencing the development of PTS/PTSD after each reproductive loss. TOP and other reproductive losses were included to provide a comprehensive account of the research literature, including a quality assessment and a direct examination of the differences between TOPS and reproductive losses in relation to PTS and PTSD.

## 2. Methods

### 2.1. Search Strategy

The electronic databases (AMED, British Nursing Index, CINAHL, MEDLINE, SPORTDiscus, PsycINFO, PubMEd, and ScienceDirect) were searched for relevant articles and followed the PRISMA guidelines [24]. No restriction for time of publication was set and only English language peer-reviewed publications were included. The search was last updated in May 2012. In PubMEd, the following key words were used in title/abstract search: “miscarriage” or “stillbirth” or “abortion” or “neonatal death” or “perinatal loss” or “failed IVF” or “failed in vitro fertilization” or “failed in vitro fertilization” or “pregnancy loss” or “termination of pregnancy” and “trauma” or “stress” or “PTSD” or “posttraumatic stress,” or “posttraumatic stress disorder.”

### 2.2. Study Selection

All papers had to be published in peer-reviewed journals, available in English and presenting original data. Studies were selected if they investigated PTSD/PTS associated with TOP and/or other reproductive losses (miscarriage, perinatal loss, stillbirth, neonatal death, and failed IVF). Quantitative studies were selected if they used standardised measurements of PTS/PTSD and qualitative studies were selected if they investigated the trauma of TOP and reproductive loss in the interviews. Studies had to use standard criteria for PTS/PTSD to be included. For PTS the reaction to traumatic events is characterised with involuntary repetition in thought, emotion, and behaviour of stress relevant contents. PTS is a marker of possibly developing the disorder, which depends on the intensity of these and other symptoms and require other conditions to become a disorder [25]. We did not include ASD in our systematic review because ASD does not necessarily lead to PTSD and is more time limited. Further, PTSD (PTS's disorder) is the development of characteristic symptoms following exposure to an extreme traumatic stressor. The characteristic symptoms resulting from the exposure to the extreme trauma include persistent reexperiencing of the traumatic event, persistent avoidance of stimuli associated with the trauma and numbing of general responsiveness, and persistent symptoms of increased arousal [26].

**Figure 1:**
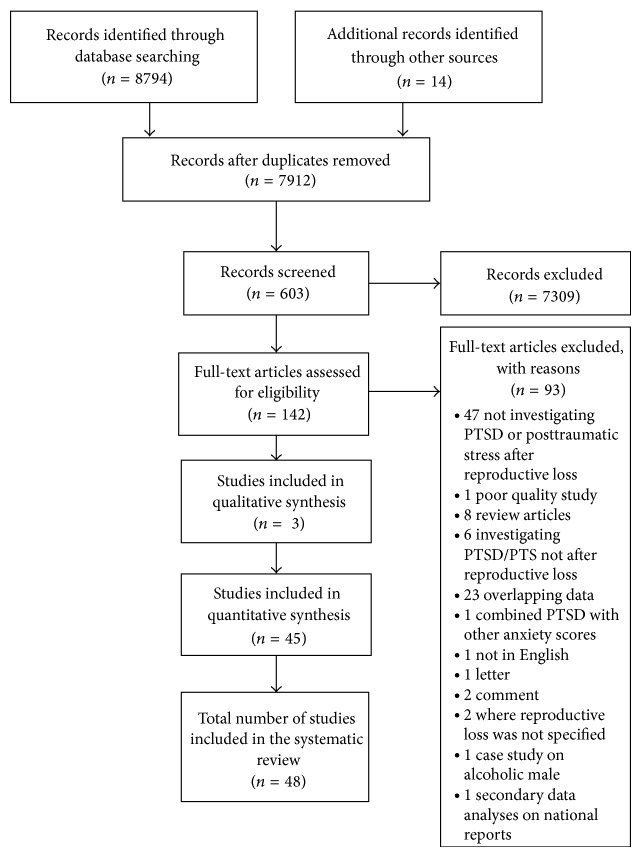
PRISMA flow diagram.

Studies which investigated existing PTS/PTSD as a risk factor for TOP or reproductive loss were excluded because the focus was on reproductive loss as a risk factor for PTS/PTSD. Further, data were collected on whether the studies included controlled for pre-TOP or prereproductive loss of mental health in their data analyses. Clinical case studies with no research agenda, books, correspondence letters, discussions, book reviews, product reviews, editorials, publisher's notes, and errata were excluded from the review. To avoid multiple publication bias [27] only one paper was selected from multiple publications and selection was based upon highest quality, followed by largest number of participants, highest number of reproductive losses, longest length of follow-up, and the paper with most reported outcome measurement data. Numbers included and reasons for exclusion are shown in Figure 1.

### 2.3. Data Abstraction

A data extraction sheet was used to collect relevant information. This included author's details, country of study, design, sample size, variables measured, results, quality of study evaluation. Data was extracted from relevant articles (VD) and cross-checked (OvdA and SP).

### 2.4. Screening and Quality Assessment

Quality assessment of articles which met the inclusion criteria was determined by VD using Cochrane criteria adapted by Green et al. [28]. These were independently checked by OvdA and SP, and disagreements were resolved following discussion; criteria were

- adequate sample size,
- representative of study population,
- high response rate,
- using mostly validated measures,
- mostly appropriate timing of measures,
- measures consistent with aims,
- conclusions consistent with results,
- methodology is clear,
- analysis is clear.

## 3. Results

As can be seen from the PRISMA flow chart (Figure 1), the search of the databases yielded 8794 titles of records and 14 records from searching reference lists. After duplicates were removed, 7912 records were left. Titles were reviewed and 7309 articles did not meet the inclusion criteria. Of the 603 abstracts reviewed, 461 failed to meet the inclusion criteria. Finally full texts of the remaining 142 papers were read and 48 studies were identified as meeting the inclusion criteria. As shown in Figure 1, 23 studies [29–51] were excluded for reporting overlapping data and 12 studies [22, 23, 52–61] from multiple reports were included. Further, one eligible study was removed [62] over serious reported methodological and statistical concerns over the study [63].

### 3.1. Study Characteristics

The study characteristics of the 48 included articles are shown in Tables 1–4; Table 1 includes TOP, Table 2 miscarriage, Table 3 perinatal loss, and Table 4 stillbirths. Each table is separated in two parts (a and b) with section a representing studies which have investigated the type of reproductive loss alone and section (b) presenting studies which have investigated that type with other losses.

As shown in Table 1, 20 studies examined PTS/PTSD after TOP (Table 1(a)); seven examined TOP with miscarriage, perinatal loss, or neonatal death (Table 1(b)). Eighteen reported nonmedical TOP [22, 23, 53, 61, 62, 64–76], one nonmedical TOP and medical TOP [77]; eight were medical TOP [58–60, 77–82]. Table 2 shows 10 studies examining PTS/PTSD after miscarriage (Table 2(a) [54, 83–91]), five miscarriages with perinatal loss, stillbirth, and neonatal death (Table 2(b) [92–96]). Table 3 shows that one study examined PTS/PTSD after perinatal loss (Table 3(a) [52]) and two studies examined perinatal loss with neonatal death (Table 3(b) [57, 97]). Finally, Table 4 reports two studies investigating PTS/PTSD after stillbirth (Table 4(a) [56, 98]) and one stillbirth with neonatal death (Table 4(b) [99]). No study investigated PTS or PTSD after failed IVF.

Some studies did not distinguish between reproductive types in their data analyses (e.g., [57, 81, 82, 93, 94, 99]) and gestational ages were not reported for *n* = 10/48 studies. The majority of studies used prospective designs, and sample sizes were generally small. PTS was consistently measured (*n* = 33/49) with the Impact of Event Scale (IES), the Revised IES (IES-R), or Perinatal Event Scale-adapted from Impact of Events Scale. The IES includes two subscales, intrusion and avoidance, and the IES-R also includes hyperarousal. Diagnoses of PTSD were done using diagnostic interviews (*n* = 4) or questionnaires (*n* = 10). Timing of outcome measurements or time since reproductive loss ranged from immediately after (*n* = 7/48) up to one year postloss (*n* = 25/48). Most quantitative studies did not control for pre-TOP or prereproductive loss of mental health parameters in their statistical analyses (33/48). Data from 6379 women and men who experienced TOP or reproductive loss and 573 controls were included in the review. The majority of studies were conducted either in Europe (*n* = 24/48) or the USA (*n* = 18/48), most participants were white, and postloss support in Western countries is likely to be better resourced than in developing countries [8]. The quality of the studies was mostly good. See the Appendix more detailed information on each study.

### 3.2. Prevalence of PTS and PTSD after TOP and Reproductive Loss

Where more than one type of reproductive loss is reported (*n* = 16/48), studies are only discussed with the TOP or reproductive loss they are presented with, as demonstrated on Tables 1–4. Further, only observational studies are presented here; intervention studies (i.e., therapy or TOP procedure type, nonsurgical versus surgical) are discussed later.

#### 3.2.1. Nonmedical TOP

For nonmedical TOP 12.6% met PTSD criteria, similar to rates for women with a prior miscarriage (12.5%) but higher than women without prior reproductive loss (6.3%) [55]. Data from online surveys estimated much higher rates of PTSD (54.9% women and 43.4% of men) up to 15 years after the TOP [22]. However, recruitment was through online resources including abortion support groups suggesting that the sample may not be representative.

Studies from abortion clinics reported moderate levels (19.4%) of PTS at two months post-TOP decreasing over time [66], and few cases of PTSD (1% *n* = 441) were reported at two years' post-TOP [23]. Adolescents also report low scores on intrusion four weeks post-TOP (719), and one study reported that PTS was high before TOP but decreased within 5 hours postoperation [65] or reported initially high PTS reducing to “negligible levels of distress” at 3 months post-TOP [64].

No differences between men and women or at 1 and 6 months were found for nonmedical TOP or medical TOP and miscarriage [77]. Before nonmedical TOP, women were less likely to report PTS intrusion than women before miscarriage (*n* = 40) and less PTS avoidance at 2 years and 5 years post-TOP compared to post miscarriage [53]. American women (14.3%) are more likely to meet full diagnostic criteria for PTSD compared to Russian women (0.9%) [72].

#### 3.2.2. Medical TOP

Following medical TOP, reports of PTS are high (64.5%) [58], with PTS reducing from 67% to 41% at 12 months. Second trimester medical TOP is more likely to result in PTS at six weeks than first trimester medical TOP but this difference disappeared at 12 months [79]. In a retrospective study of medical TOP, 33% continued to report PTS up to a mean time of 4 years since the loss [59]. Women (44%) report higher rates of PTS than men (21.6%) [60]; pregnant women who had a previous medical TOP are less likely to report PTS than pregnant women with previous miscarriage [78] and significantly lower PTS is reported after medical TOP than perinatal/neonatal death [82].

#### 3.2.3. Miscarriage

One study found that PTSD is infrequently reported three months after miscarriage [90]. Of studies reporting PTS after miscarriage, a reduction is reported after 3 [91] to 4 months [54], although an increase in PTSD over time has also been reported [84]. Other reports find higher levels with 67.9% of pregnant women with prior miscarriage or perinatal loss meeting partial or full criteria for PTSD [93]. Similar high numbers (82% PTS; 80% PTSD) were reported in men and women after miscarriage, perinatal, stillbirth, or neonatal death/infant death three years previously [94]. However, data was not separated for reproductive loss type and the sample was recruited from a support group and may not be representative. Hospital samples record lower numbers (11% with PTSD) which reduced still further (2.8%) 4–12 months follow-up [95].

Women who experienced recurrent miscarriage were more likely to report intrusion [83] or intrusion and avoidance than their partners [89]. Pregnant women who had a previous miscarriage or perinatal loss scored high on avoidance and men scored high on intrusion with 88% of women and 90% of men meeting the cut-off for PTS [92], confirming other reports of clinical levels of PTS in men [85].

#### 3.2.4. Perinatal Loss

PTS is initially high [52, 97] and decreases to moderate levels 8 months postpartum in couples with a history of perinatal loss who subsequently had a healthy infant [52]. Specifically intrusion increased more in women and avoidance remained stable in both [52], or PTS remained high and unchanged from the first diagnosis to three months after delivery/death [57].

#### 3.2.5. Stillbirth

One longitudinal study of 65 pregnant women who had a prior stillbirth reported 21% PTSD in the third trimester and 4% at one year after birth [56].

### 3.3. Factors Influencing PTS and/or PTSD after TOP and Reproductive Loss

#### 3.3.1. Nonmedical TOP

Having a TOP predicted a diagnosis of PTSD and sociodemographic variables (younger age, poverty, poor education, poor housing, and race), history of sexual trauma, and illness or medical trauma are other risk factors independently predicting PTSD [55]. A history of sexual or medical trauma doubled the risk for PTSD [55], whereas harsh discipline as a child, adult rape, and physical or emotional abuse were associated with PTSD [72], and a history of major depression also predicted PTSD [23]. Peritraumatic dissociation and difficulties in describing feelings were significant predictors of PTS [66].

Relationship stability as a reason to continue the pregnancy is a strong predictor of intrusion symptoms [64]. Couple's disagreement towards having a TOP and inadequate before abortion counselling significantly predicted PTSD in women and men [22] and in women, knowing others who have not coped with TOP [64], attachment to the foetus, recognition of life, time since abortion, and increased maternal age predicted PTSD [61]. Higher levels of perceived quality in couple's relationship [77] and active coping influence short term PTS after nonmedical TOP [65]. Spiritual group therapy reduces PTS for women experiencing post-TOP grief, although no control group was used [69] and hypnosis with psychological therapy successfully reduced symptoms of PTSD in a case study [76]. Recurrent thoughts continued to affect and traumatise women's lives up to 15 years post-TOP in a qualitative study [75].

Research considering type of TOP procedure reporting nonsurgical TOP predicts PTSD [68, 71], surgical TOP is associated with PTS [67], or no differences between nonsurgical or surgical TOP on PTS [72]. No differences between local anaesthesia and intravenous sedation in surgical TOP were reported either [74].

#### 3.3.2. Medical TOP

PTS in women was also predicted by sociodemographic factors [79] (low education, younger maternal age, and advanced gestation) and low levels of partner support [59, 60], whereas, for men, being religious and doubt over decision predicted PTS [60]. PTS is also associated with perinatal grief, depression, and anxiety for pregnant women with prior medical TOP or miscarriage [78]. In women with medical TOP, miscarriage, stillbirth, and neonatal death, recruited through support groups, PTS was correlated with low levels of social support, perinatal grief, emotional pain, emotional expression, and, less strongly, dream frequency [81]. Depression had also been associated with high intrusion scores for women who experienced medical TOP and perinatal loss/neonatal death [82]. Difficult physical symptoms of miscarriage and TOP and having to make the decision to have a medical TOP were experienced as traumatic in a qualitative investigation [80].

#### 3.3.3. Miscarriage

A diagnosis of acute stress disorder [84], peritraumatic dissociation, and neuroticism [54] leads to PTSD one to 4 months after loss. Unplanned pregnancies are significantly related to PTS [91]. Both men and women have clinical levels of PTS although sociodemographic variables and quality of relationship do not predict PTS for either [89]. However, for men, PTS was more likely to be associated with perinatal grief and older gestational age and viewing the ultrasound scan were significantly associated with PTS [85], whereas, for women, viewing scans and early warning signs for miscarriage were not associated with PTS [91]. Psychological therapy to reduce PTS is ineffective; it usually declines spontaneously over time [86–88].

Depression, and pregnancy related anxiety, but not prenatal attachment related to PTS in couples with prior miscarriage or perinatal loss [92]. Depression, anxiety, and poly substance disorders cooccurred for some women with full or partial PTSD diagnosis after miscarriage and other reproductive losses [93]. PTSD is also associated with feelings of doubt [94] and attribution of blame [95]. Ultrasound of the current pregnancy triggered flashbacks and symptoms of PTSD for some women with prior miscarriage, perinatal loss, stillbirth, and infant loss [96].

#### 3.3.4. Perinatal Loss

Depression was significantly correlated with PTS during a current pregnancy and eight months following delivery for women and men with prior perinatal losses [52]. For women, anxiety was associated with PTS during pregnancy but, for men, anxiety was associated with PTS during and after delivery [52]. For pregnant women with a history of perinatal loss and neonatal death, intrusion was associated with an increase of women's healthcare use [97]. For women experiencing a neonatal death, those who delivered early were more likely to experience intrusion than women who delivered after 34 weeks [57].

#### 3.3.5. Stillbirth

At one year postdelivery, seeing and holding the stillborn infant was significantly associated with PTSD in pregnant women with a previous history of stillbirth [56]. Attending support groups significantly predicted lower PTS [98]. Finally, in women who experienced a stillbirth or neonatal death, intrusion and hyperarousal predicted perinatal grief [99].

## 4. Conclusion

Systematic research evidence on the prevalence of PTS or PTSD associated with failed IVF is nonexistent, and few studies reported on PTS/PTSD following perinatal loss, neonatal death, or stillbirth, usually alongside other reproductive losses. There were more studies on PTS/PTSD after miscarriage and TOP for nonmedical and medical reasons. However, the research is inconsistent with regards to prevalence rates which depended on how participants were recruited. In some cases, no prevalence rates, reproductive loss type, or gestational age of the loss were recorded, reflecting the lack of research into the mental health of patients following reproductive loss [18].

Overall, this review has demonstrated that PTSD occurs after nonmedical and medical TOP, miscarriage, perinatal loss, and stillbirth, although it is much less commonly reported than PTS. Length of gestational age is associated with an increased likelihood for diagnosis of PTS or PTSD. The percentage of PTS and its disorder is highest during the first weeks after TOP or reproductive loss and decreases significantly over time for most but not all women and men. Women generally report more PTS or PTSD symptoms but clinical levels of distress are also reported for men.

Research has generally demonstrated that PTS or PTSD after TOP and reproductive losses are complex and a variety of factors play an influencing role. Studies which have investigated the impact of sociodemographic characteristics (TOP and miscarriage studies) and the experience of other previous traumas on PTS and PTSD have found that demographic factors such as maternal age, gestational age, lower education, and a history of previous physical or sexual trauma are significant risk factors for the development of PTS or PTSD after loss. Prior history of mental health problems and current depression, anxiety, and perinatal grief are also risk factors, confirming previous research [100], although it is not clear if the mental health or the known lack of health seeking behaviour is responsible for the reproductive loss [101]. Time has generally been found to be the most influential protective factor in reducing levels of PTS/PTSD. The evidence for the effectiveness of “individual” psychological therapy is mixed and generally suggest therapy is ineffective at reducing PTS or PTSD anymore than time does by itself. The quality of relationship between the couple has also been found to act as a protective factor, as is found more generally in research reporting coping with reproductive disorders [3].

The quality of studies included in the review was generally good, reaching average scores of 7/9, but the samples are often small, select, and nonrepresentative. Most studies report data for one year after TOP or loss and no inferences can be drawn about the long term consequences. This is a serious limitation because the time since the loss occurred is an important factor that influences PTS and PTSD. Further, most of the studies included did not control for pre-TOP or prereproductive loss mental health in their statistical analyses. This is potentially another significant limitation of existing research because a recent Danish population based cohort study found evidence that the incidence of pre-TOP psychiatric contacts up to 9 months preabortion (14.6%) was similar to the incidence at 12 months post-TOP (15.2%) among the large cohort of girls and women included in the analysis [102]. The lack of research on perinatal loss, stillbirth, and neonatal death is also of concern because a longer gestation predicts an increasing likelihood of PTS or PTSD. Evidence of PTS and reproductive loss of men, nonwhite, single women, adolescent girls, and women past the age of natural childbearing is meagre and nonexistent on failure of infertility treatment needing further attention. Finally, most of the studies that have examined TOP have come from countries that permit nonmedical TOP. Restrictive TOP laws are not associated with lower TOP rates, indeed the opposite appears to be true [13, 14]. Therefore, the findings from this review cannot easily be transferred to those countries where TOP is illegally practiced, particularly where unsafe TOP's are carried out.

To sum, this systematic review investigated PTS/PTSD after TOP and reproductive loss. The prevalence of PTS was greater than PTSD and both decreased over time. However, the more advanced the pregnancy is, the more PTS and PTSD are likely to be reported. Women generally report more PTS/PTSD but men also report clinical rates of PTS/PTSD. Time since TOP and loss, demographic characteristics and psychosocial factors influence the development and maintenance of PTS and PTSD after TOP and reproductive loss.

## Figures and Tables

**Table 1:** Characteristics of included studies for TOP (a) and TOP with miscarriage/perinatal loss/neonatal death (b).

Authors, year, country, and author listing in reference list	Methods	Reproductive loss, Participants, and control for Prereproductive loss	Outcomes measurements	Quality
(a) Termination of pregnancy (TOP). Numbers of participants are women, men, or couples and are noted separately.				

(1) Allanson (2007), Australia [64]	P, L	Nonmedical TOP *n* = 96 **Prereproductive loss mental health parameters statistically not controlled**	IES	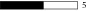

(2) Cohen and Roth (1984), USA [65]	P	Nonmedical TOP *n* = 55 **Prereproductive loss mental health parameters statistically controlled**	IES	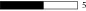

(3) Coyle et al. (2010) USA [22] *Note. This study was chosen over [32]; it had larger number of reported participants. *	R	Nonmedical TOP 374 women; 198 men **Prereproductive loss mental health parameters statistically controlled**	PTSD Checklist-Civilian Version	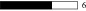

(4) Davies et al. (2005), UK [79]	P	Medical TOP 30; **Prereproductive loss mental health parameters statistically not controlled**	IES	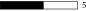

(5) van Emmerik et al. (2008), The Netherlands [66]	P	Nonmedical TOP 67 **Prereproductive loss mental health parameters statistically not controlled**	IES	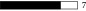

(6) Hemmerling et al. (2005), Germany [67]	P, I	Nonmedical TOP 219 **Prereproductive loss mental health parameters statistically not controlled**	IES	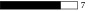

(7) Kelly et al. UK (2010) [68]	P, I	Nonmedical TOP 122 **Prereproductive loss mental health parameters statistically controlled**	IES	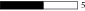

(8) Kersting et al. (2009), Germany [58] *Note. This study was chosen over [41] because of a higher quality rating and [42] because it reports more participants and relevant data. *	P, L	62 medical TOP; 43 preterm birth; 65 spontaneous delivery **Prereproductive loss mental health parameters statistically not controlled**	IES-R	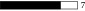

(9) Korenromp et al. (2005a), The Netherlands [59] *Note. This study was chosen over [43] because of higher quality rating. *	R	Medical TOP 196 **Prereproductive loss mental health parameters statistically not controlled**	IES	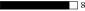

(10) Korenromp et al. (2007), The Netherlands [60] *Note. This study was chosen over [44] because of a larger sample. *	P, L	Medical TOP 217 women; 169 men **Prereproductive loss mental health parameters statistically not controlled**	IES	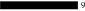

(11) Layer et al. (2004), USA [69]	P, I	Nonmedical TOP 35 **Prereproductive loss mental health parameters statistically not controlled**	IES	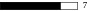

(12) Major et al. (2000), USA [23] *Notes. This study was chosen over [45] because it reported more data. *	P, L	Nonmedical TOP 442 **Prereproductive loss mental health parameters statistically not controlled** for PTSD	Adapted PTSD measure-using DSM-III-R-used with Vietnam War veterans	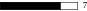

(13) Mufel et al. (2002), Belarus, USA [61] *Note. This study was chosen over *[*475 *]* because of a higher quality rating. *	R	Nonmedical TOP 150 **Prereproductive loss mental health parameters statistically not controlled**	IES-R	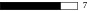

(14) Pope et al. (2001), USA [70]	P	Nonmedical TOP 96 **Prereproductive loss mental health parameters statistically not controlled** for PTS	IES	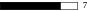

(15) Rousset et al. (2012), France [71]	P, I	Nonmedical TOP 70 **Prereproductive loss mental health parameters statistically not controlled**	IES-R	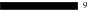

(16) Rue et al. (2004), USA [72]	R	Nonmedical TOP 331 Russian women; 217 American women **Prereproductive loss mental health parameters statistically not controlled**	Institute of Pregnancy Loss questionnaire-including criteria for PTSD on DSM-IV	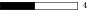

(17) Slade et al. (1998), UK [73]	P, I	Nonmedical TOP 275 **Prereproductive loss mental health parameters statistically not controlled** for PTS	IES	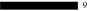

(18) Suliman et al. (2007), South Africa [74]	P, I	Nonmedical TOP 151 **Prereproductive loss mental health parameters statistically controlled**	Clinician-administered PTSD scale (CAPS-I)	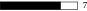

(19) Trybulski (2006), USA [75]	Q	Nonmedical TOP 16	Qualitative interview.	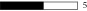

(20) Walters and Oakley (2002), UK [76]	CS	Nonmedical TOP 1 **Prereproductive loss mental health parameters statistically not controlled**	The Post-Traumatic Stress Diagnostic Scale	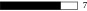

(b) TOP and miscarriage/perinatal loss/neonatal death				

(21) Broen et al. (2005b), Norway [53] *Note. This study was chosen over [31]; it has a higher quality rating/longer follow-up time [30]. *	P, L	40 miscarriages; 80 Nonmedical TOP **Prereproductive loss mental health parameters statistically controlled**	IES	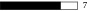

(22) Canário et al. (2011), Portugal [77]	P	Nonmedical TOP (30); medical TOP (10); miscarriage (10). **Prereproductive loss mental health parameters statistically not controlled**	IES-R	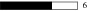

(23) Cowchock et al. (2011), USA [78]	P	7 medical TOP, 8 miscarriages **Prereproductive loss mental health parameters statistically not controlled**	IES	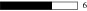

(24) Fernandez et al. (2011), Canada [80]	Q	2 medical TOP; 5 miscarriages	Qualitative interviews	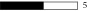

(25) Hamama et al. (2010), USA [55] *Note. This study was chosen over [46] because of more relevant PTSD data. *	P	405 prior pregnancies; 221 prior nonmedical TOP; 206 miscarriages; 22 reported both **Prereproductive loss mental health parameters statistically not controlled**	Interview (National Women's Study PTSD Module (NWS-PTSD)).	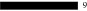

(26) Kroth et al. (2004), USA [81]	R	Medical TOP, miscarriage, perinatal loss, and neonatal death 37 women **Prereproductive loss mental health parameters statistically not controlled**	IES	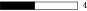

(27) Salvesen et al. (1997), Norway [82]	P	24 medical TOP, 29 perinatal losses/neonatal deaths **Prereproductive loss mental health parameters statistically not controlled**	IES	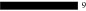

Notes: CS = case study; I = intervention design; IES = Impact of Event Scale; IES-R = Revised; L = longitudinal; P = prospective; Q = qualitative; R = retrospective.

**Table 2:** Characteristics of included studies for miscarriage (a) and miscarriage with perinatal loss/stillbirth/neonatal death (b).

Authors year, country, and author listing in reference list	Methods	Reproductive loss, Participants, and control for Prereproductive loss	Outcomes measurements	Quality
(a) Characteristics of included studies for miscarriage				

(28) Alderman et al. (1998), USA [83]	R	Miscarriage 19 couples **Prereproductive loss mental health parameters statistically not controlled**	IES	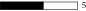

(29) Bowles et al. (2006), USA [84]	P, L	Miscarriage 25 **Prereproductive loss mental health parameters statistically not controlled**	Posttraumatic Stress Diagnostic Scale	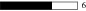

(30) Engelhard et al. (2003a), The Netherlands [54] *Note. This study was chosen over [33–37] because of the largest number of reported participants/reported the most data. *	P, L	Miscarriage 118 **Prereproductive loss mental health parameters statistically controlled**	Posttraumatic Symptom Scale	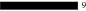

(31) Johnson and Puddifoot (1996), UK [85]	P	Miscarriage 126 men **Prereproductive loss mental health parameters statistically not controlled**	IES	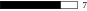

(32) Lee et al. (1996), UK [86]	P, I	Miscarriage 39 **Prereproductive loss mental health parameters statistically controlled**	IES	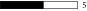

(33) Rowsell et al. (2001), UK [87]	P, I	Miscarriage 37 **Prereproductive loss mental health parameters statistically not controlled**	IES	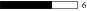

(34) Séjourné et al. (2010), France [88]	P, I	Miscarriage 134 **Prereproductive loss mental health parameters statistically not controlled for PTS**	IES-R	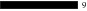

(35) Serrano and Lima (2006), Portugal [89]	R	Miscarriage 30 women and 30 men **Prereproductive loss mental health parameters statistically not controlled**	IES	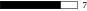

(36) Sham et al. (2010), Hong Kong [90]	P, L	Miscarriage 161 **Prereproductive loss mental health parameters statistically controlled **	Structural clinical interview for DSM-IV	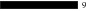

(37) Walker and Davidson (2001), UK [91]	P	Miscarriage 40 **Prereproductive loss mental health parameters statistically controlled**	IES	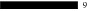

(b) Miscarriage and perinatal/stillbirth/neonatal death				

(38) Armstrong (2004), USA [92]	P	Miscarriage and Perinatal loss 40 expectant couples **Prereproductive loss mental health parameters statistically not controlled**	IES	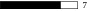

(39) Forray et al. (2009), USA [93]	P	Miscarriage; perinatal loss/neonatal loss/other complications 76 pregnant women, of which 18 underwent interviews **Prereproductive loss mental health parameters statistically not controlled**	(Modified Clinical administered PTSD Scale (m-CAPS))	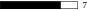

(40) Jind (2001), Denmark [94]	R	Miscarriage/perinatal, stillbirth, and neonatal death/infant loss 602 parents **Prereproductive loss mental health parameters statistically not controlled**	IES, Harvard trauma Questionnaire	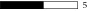

(41) Jind (2003), Denmark [95]	P, L	Miscarriage, perinatal loss, stillbirth, and neonatal death/infant loss; 93 parents at the first measurement, 65 parents at the second measurement **Prereproductive loss mental health parameters statistically not controlled**	The Harvard Trauma Questionnaire	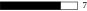

(42) O‘leary (2005), USA [96]	Q	Miscarriage, perinatal loss, stillbirth and infant loss; 12 expecting mothers and 9 expecting fathers	Qualitative Interviews.	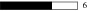

**Table 3:** Characteristics of included studies for perinatal loss (a) and perinatal loss with neonatal death (b).

Authors year, country, and author listing in reference list	Methods	Reproductive loss, Participants and control for Prereproductive loss	Outcomes measurements	Quality
(a) Perinatal loss				

(43) Armstrong et al. (2009), USA [52] *Note. This study was chosen over [29]because it had a higher quality rating. *	P, L	Perinatal loss 36 couples **Prereproductive loss mental health parameters statistically not controlled**	IES	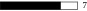

(b) Perinatal loss and neonatal death				

(44) Hunfeld et al. (1993), Netherlands [57] *Note.This study was chosen over [39, 40] because of a higher quality rating and [38] because of more relevant PTSD data. *	P, L	Perinatal loss and neonatal death; 46 **Prereproductive loss mental health parameters statistically not controlled**	Perinatal Event Scale –adapted from Impact of Event Scale	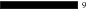

(45) Hutti et al. (2011), USA [97]	P, L	Perinatal loss and neonatal death; 106 women **Prereproductive loss mental health parameters statistically not controlled**	IES	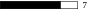

**Table 4:** Characteristics of included studies for stillbirth (a) and stillbirth with neonatal death (b).

Authors year, country, and author listing in reference list	Methods	Reproductive loss, Participants and control for Prereproductive loss	Outcomes measurements	Quality
(a) Stillbirth				

(46) Cacciatore (2007), USA [98]	R	Stillbirth 47 **Prereproductive loss mental health parameters statistically not controlled**	IES-R	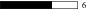

(47) Hughes et al. (2002), UK [56] *Note. This study was chosen over [48–51] because of the highest quality rating/reported more participants/data. *	P	65 pregnant women, with prior stillbirth, 60 controls **Prereproductive loss mental health parameters statistically controlled**	PTSD-1 Interview	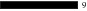

(b) Stillbirth and neonatal death				

(48) Uren and Wastell (2002), Australia [99]	R	Stillbirth and neonatal loss; 109 women **Prereproductive loss mental health parameters statistically not controlled**	IES-R	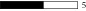

## Appendix

See Tables 1, 2, 3, and 4.
